# Antibody Surface Coverage Drives Matrix Interference in Microfluidic Capillary Immunoassays

**DOI:** 10.1021/acssensors.1c00704

**Published:** 2021-06-17

**Authors:** Ana I. Barbosa, Alexander D. Edwards, Nuno M. Reis

**Affiliations:** † Department of Chemical Engineering, Loughborough University, Loughborough LE11 3TU, United Kingdom; ‡ Capillary Film Technology Ltd, Daux Road, Billingshurst RH14 9SJ, West Sussex, United Kingdom; § Reading School of Pharmacy, University of Reading, Whiteknights, Reading RG6 6AD, United Kingdom; ∥ Department of Chemical Engineering and Centre for Biosensors, Bioelectronics and Biodevices (C3Bio), University of Bath, Claverton Down, Bath BA2 7AY, United Kingdom

**Keywords:** matrix effect, microfluidics, biosensors, protein biomarkers, microcapillary film

## Abstract

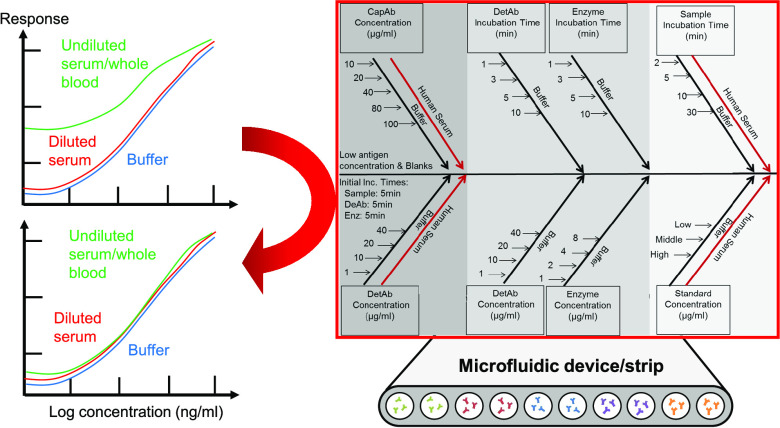

The performance of biosensors is often optimized in buffers, which brings inconsistencies during applications with biological samples. Current strategies for minimizing sample (matrix) interference are complex to automate and miniaturize, involving, e.g., sample dilution or recovery of serum/plasma. This study shows the first systematic analysis using hundreds of actual microfluidic immunoassay fluoropolymer strips to understand matrix interference in microflow systems. As many interfering factors are assay-specific, we have explored matrix interference for a range of enzymatic immunoassays, including a direct mIgG/anti-mIgG, a sandwich cancer biomarker PSA, and a sandwich inflammatory cytokine IL-1β. Serum matrix interference was significantly affected by capillary antibody surface coverage, suggesting for the first time that the main cause of the serum matrix effect is low-affinity serum components (e.g., autoantibodies) competing with high-affinity antigens for the immobilized antibody. Additional experiments carried out with different capillary diameters confirmed the importance of antibody surface coverage in managing matrix interference. Building on these findings, we propose a novel analytical approach where antibody surface coverage and sample incubation times are key for eliminating and/or minimizing serum matrix interference, consisting in bioassay optimization carried out in serum instead of buffer, without compromising the performance of the bioassay or adding extra cost or steps. This will help establishing a new route toward faster development of modern point-of-care tests and effective biosensor development.

Components of biological samples are known to interfere with the performance of diagnostics tests, by affecting the response of the test to the analyte of interest.^1^ This has a direct impact on sensitivity, specificity, and variability of the test, leading to inaccurate analyte quantitation in real biological samples.^2,3^ According to the current literature, this so-called matrix interference or effect can be caused by different components; blood cells, sample viscosity, or plasma components such as heterophilic antibodies (antibodies produced against poorly defined antigens presenting low affinity and weak binding),^4^ human antianimal antibodies (HAAA, high-affinity antibodies generated when the immune system is in contact with animal antibodies),^5^ and other plasma proteins such as albumin, lysozyme, fibrinogen, and paraprotein have been reported to cause test interferences.^6^ Boscato et al. showed that analyte–antibody binding substances were detected in 40% of studied samples (688 samples), being responsible for 15% interference in assays.^7^ Appropriate matrix management is therefore essential to develop reliable bioassays and biosensors; however, this is highly dependent on the molecular analysis platform since the type of reagents (e.g., antibody purity) and the antibody binding conditions (e.g., antibody affinity, diffusion distance, surface interactions) are key contributors to the matrix effect. Although current procedures for dealing with matrix interference can be effectively implemented in a centralized pathology lab, involving conventional sample preparation methods such as dilution, centrifugation, precipitation, etc., these methods are not universal and fail to serve sensitive and automated detection desired in portable point-of-care (POC) microfluidic platforms.^8^ Currently, little is known in the literature about matrix interference in microfluidic systems, which needs to be addressed to speed up the adoption of microfluidic bioassays and biosensors.

To find a universal way to deal with the matrix effect at POC settings, there are a plethora of microfluidic plasma separation devices aiming to eliminate sample matrix interference in protein bioassays performed by novel biosensor platforms.^9,10^ However, plasma or serum still contains interfering factors, which affect the accuracy of the tests.^11^ POC analytical approaches would greatly benefit from overcoming biological matrix interference without laboratory equipment, since any sample preparation required for a POC test compromises the speed, complexity, and cost of the test. Therefore, understanding the biological matrix interference and finding strategies to overcome it are paramount for the POC diagnostic industry and biosensor research,^12^ which aim to combine sensitive, accurate, and rapid protein quantitation with cost-effective test development, demanded by the ever-increasing biomarker use in patients’ stratification and personalized medicine.^3,8,13,14^ Many biosensors are incorporated into lab-on-a-chip devices that test plasma or serum separated outside the microdevice using centrifugation, reducing the benefits of miniaturization.^15^ A growing number of microfluidic strategies aim to incorporate *in situ* plasma separation from whole blood^14^ using microstructures,^16^ gravity-driven separation,^17^ microcentrifugation,^18^ capillary-driven contactless electrophoresis,^19^ and the plasma skimming effect sometimes referred to as the Zweifach–Fung effect.^20,21^ However, very few studies reported the measurement of protein biomarkers after the blood plasma separation, which hinders the validation of the developed devices and methods for protein biomarker quantitation. Furthermore, the microfluidic studies that actually report protein detection in plasma^22,23^ do not report recovery or sample variability studies, hampering the understanding of how blood or plasma sample matrix affects protein biomarker detection in microfluidic devices and consequently how to solve the sample variability effect. In fact, data that reflects how sample components affect antibody–antigen binding in a specific microfluidic device can be difficult to obtain, not only due to the variety of interference factors but also due to the prototype nature of microfluidic devices that are not manufactured on a large scale, reducing the number of replicates needed for the study. Several studies use real-time antibody–antigen detection techniques such as optical waveguide lightmode spectroscopy (OWLS), ellipsometry, or quartz crystal microbalance (QCM) that, although very precise for antibody binding affinity determination, use polymer-coated specific surfaces that not always replicate the surface chemistry of the actual microfluidic devices. Also, these systems do not reflect the geometry of the microfluidic devices, which can lead to errors when translating assay conditions from real-time detection technique to microfluidic systems.^24,25^

In the present work, we explored matrix interference in microfluidic protein immunoassays using hundreds of fluorinated, Teflon FEP microfluidic strips fabricated from a melt-extruded, mass-manufactured 10-bore microcapillary film (MCF), connected to a multiple syringe aspirator developed in-house (Figure 1A). Microfluidic protein bioassays presented significant variations when performed in buffer or human serum (Figure 1B), confirming that matrix interference is also present in microfluidic bioassays. Based on our previous experience in carrying out high-performance immunoassays in this microfluidic platform,^3,26^ we hypothesized that the actuation mechanism of the interfering factor(s) (Figure 1C) is closely related to the antibody surface coverage. Consequently, in this study we explored the impact of antibody surface coverage on sample matrix interference for three distinct protein bioassays, as interference can be very assay-specific.^27,28^ In addition, we studied other parameters that appear to contribute to the matrix interference, with a particular focus on sample incubation time and capillary diameter. We gathered the outcomes into a new bioanalytical approach for minimizing matrix interference in immunoassay protein detection.

**Figure 1:**
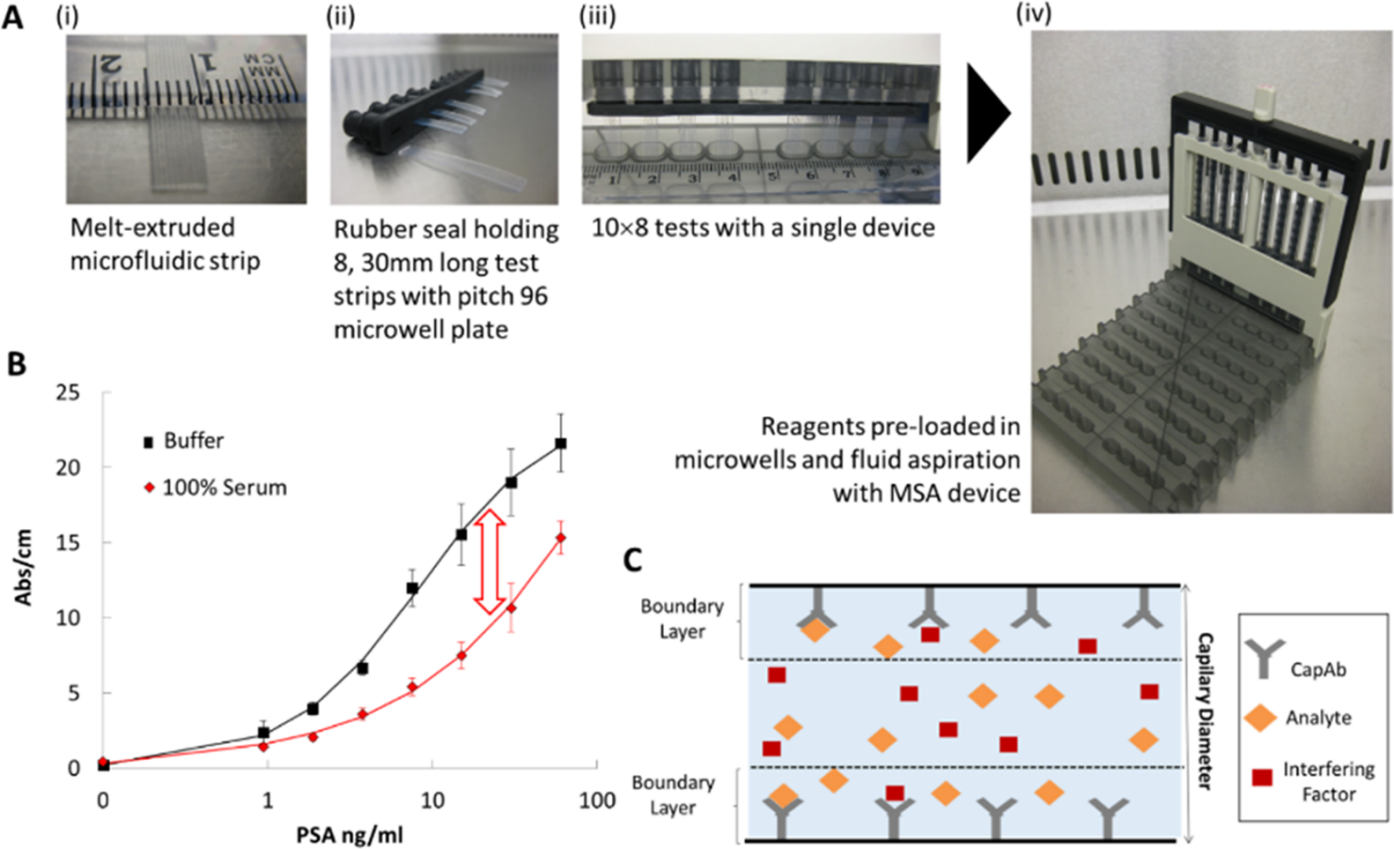
Human serum matrix effect in MCF diagnostic strips. (A) MCF and the fluid handling setup for diagnostic procedures. (B) PSA sandwich assay full response curves in human serum and buffer, showing the matrix effect interference. (C) Schematic of the capillary immunoassays in the MCF platform.

## Experimental Section

### Materials and Reagents

Mouse IgG (mIgG, whole antibody) was purchased from Life Technologies (Paisley, U.K.); rabbit anti-mIgG (whole molecule) conjugated with peroxidase and SIGMAFAST OPD (*o*-phenylenediamine dihydrochloride) tablets was purchased from Sigma-Aldrich (Dorset, U.K.). The IL-1β recombinant protein, anti-Human IL-1β biotin, and anti-Human IL-1β (purified) were obtained from eBiosciences (Hatfield, U.K.). High-sensitivity streptavidin-HRP was supplied by Thermo Scientific (Lutterworth, U.K.). Human kallikrein 3/prostate specific antigen (PSA) ELISA kit was purchased from R&D Systems (Minneapolis). The kit contained a monoclonal mouse Human Kallikrein 3/PSA antibody (CapAb), a Human Kallikrein 3/PSA polyclonal biotinylated antibody (DetAb), and recombinant Human Kallikrein 3/PSA (standard). Phosphate-buffered solution (PBS) and bovine serum albumin (BSA) were sourced from Sigma-Aldrich (Dorset, U.K.). PBS, pH 7.4, 10 mM was used as the main experimental buffer. The blocking solutions consisted of 3% w/v protease-free BSA diluted in PBS buffer and a SuperBlock blocking solution purchased from Thermo Scientific (Lutterworth, U.K.). For washings, PBS with 0.05% v/v Tween-20 (Sigma-Aldrich, Dorset, U.K.) was used. A female human serum sample was supplied by BBI solutions (Cardiff, U.K.), aliquot, and stored at −20 °C. Human blood, supplied by healthy volunteers at Loughborough University, was collected into a 5 mL vial with citrate phosphate dextrose (CPD) as the anticoagulant.

### Microfluidic Fluoropolymer MCF Strips

The microengineered MCF material (materials and geometry detailed in the Supporting Information, SI)^29,30^ is particularly well suited to study systematically the role of the sample matrix on heterogeneous immunoassays, enabling simple and rapid manufacturing of hundreds or thousands of disposable strips under very identical conditions at a negligible cost, which would be hard to match with other microfluidic devices. Also, the whole inner section of the cylindrical/elliptical capillaries is homogeneously coated with the capture antibody in contrast to immobilization on a single surface as it happens for many other microfluidic devices, which offers advantages in studying surface coverage and specific/nonspecific surface binding.^31^

### Effect of Antibody Surface Coverage

To understand how antibody surface coverage influences human serum interference in MCF protein tests, three different assays (mIgG/anti-mIgG, IL-1β, and PSA assay), presenting different analytical antibodies, were studied in a 10-bore, 212 μm mean internal diameter MCF. The antibody surface coverage of these assays was varied by loading captured antibody solutions in the range of 0–200 μg/mL. The antigen concentration was kept constant in the three assays, being 0.6 μg/mL, 0.125 ng/mL, and 3.75 ng/mL for peroxidase-conjugated anti-mIgG, IL-1β, and PSA, respectively, as well as the antigen/sample incubation time was fixed at 5 min. IL-1β and PSA assays follow the same conditions as previously reported^3,32^ and briefly described in the SI. Digital images of MCF strips in the three studied assays were taken after 5 min of OPD enzymatic substrate loading. The described assays were performed in the exact same conditions preparing antigen solutions in buffer and in nondiluted human serum (refer to the SI document for more details).

### Effect of Sample Incubation Time

To better understand the sample incubation effect in the matrix interference in MCF protein assays, an IL-1β sandwich assay^32^ was performed in nondiluted human serum, whole blood, and buffer. Instead of full response curves, only four IL-1β concentrations were tested (0, 0.03, 0.125, and 0.5 ng/mL). The sandwich assays were performed with 5 and 30 min of sample incubation. To plot the IL-1β full response curve, a solution of 40 μg/mL of anti-IL-1β CapAb was used as the coating solution in a 212 ± 16 μm diameter MCF, and 1:2 serial dilutions of 0–1 ng/mL range of IL-1β were spiked in buffer, 50% serum, and 100% serum as sample diluents. The samples were incubated for 5 and 30 min. The 4-parameter logistic (4PL) mathematical model was fitted to the experimental data by the minimum square difference for each full IL-1β response curve. The lower limit of detection was calculated by the mean absorbance of the blank plus three times the standard deviation of the blank samples.

### Effect of Capillary Diameter

Several transversal sections of three FEP MCFs with different capillary diameters were trimmed, and a long focal distant point microscope (Nikon SMZ 1500 stereo microscope) was used for imaging. ImageJ software (NIH, Maryland) was used to measure the diameter of the 10 capillaries from the microphotographs.^33^ A solution of 200 μg/mL of mIgG was filled into MCF strips of three different diameters (109, 212, and 375 μm) with 35 cm length each. A negative control strip was filled with PBS buffer. The solutions were incubated for 30 min at room temperature and washed with 1 mL of PBS-Tween. A solution of 0.6 μg/mL peroxidase-conjugated mouse anti-mIgG, prepared in PBS buffer, was added to the MCF strip and 4 cm long strips were trimmed and individually washed with PBS-Tween after variable incubation times of anti-mIgG. OPD substrate (1 mg/mL) was added to the strips, and digital images were taken with a flatbed scanner after 5 min of enzymatic substrate incubation. The procedure was repeated for 0.6 μg/mL peroxidase-conjugated anti-mIgG solutions prepared in nondiluted human serum.

### Kinetics of Antibody–Antigen Binding

Equation 1 was solved analytically for a constant analyte concentration and used to estimate the rates of association and dissociation of antibody binding in the MCF system.^34^
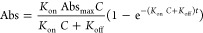
1where Abs is the optical absorbance signal corresponding to the antigen surface density at time *t*; *C* is the antigen bulk concentration; *K*_on_ is the association rate and *K*_off_ is the dissociation rate; and Abs_max_ is the maximum Abs signal corresponding to the maximum antigen surface coverage.

### Image Analysis of the Microfluidic MCF Strips

RGB digital images of the immunoassay strips were split into three separated channel images by ImageJ software (NIH, Maryland). The blue channel images were used to calculate Abs values, based on the gray-scale peak height of each individual capillary of Teflon FEP MCF, as described previously.^3,29,35^ Therefore, the absorbance signal is calculated for each capillary, according to the Beer–Lambert equation. The absorbance values presented averages of absorbance from 10 capillaries in a given MCF strip.

## Results and Discussion

### Matrix Interference is Linked to Antibody Surface Coverage

**Figure 2:**
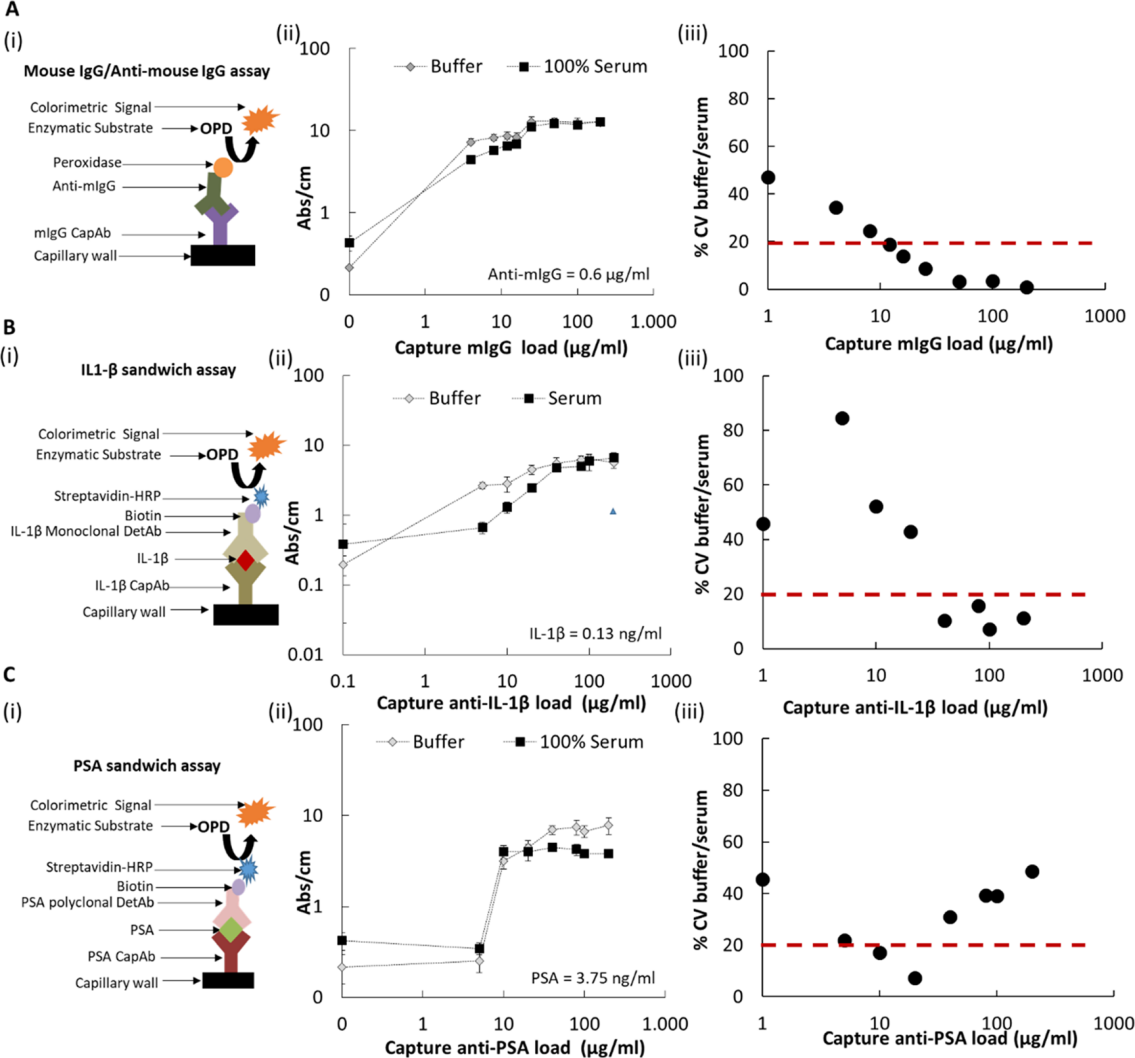
Effect of antibody surface coverage on the matrix effect of human serum of three different MCF protein assays. (A) Effect of human serum on anti-mIgG detection using a range of 0–200 μg/mL of capture antibody loading. Antigen concentration was kept constant, anti-mIgG = 0.6 μg/mL. (B) Effect of human serum on IL-1β detection using a range of 0–200 μg/mL of capture antibody loading. Antigen concentration was kept constant, IL-1β = 0.13 ng/mL. (C) Effect of human serum on PSA using a range of 0–200 μg/mL of capture antibody loading. Antigen concentration was kept constant, PSA = 3.75 ng/mL. (i) Assay schematics; (ii) shows the assay signal in buffer and serum, while (ii) shows the ratio of the two signals. The red dashed line indicates the limit of 20% variation above which the variability is not acceptable for immunoassay performance.

It has been previously shown that antibody surface coverage is related to immobilized antibodies’ functionality since it interferes with their orientation and steric hindrance.^31^ Therefore, we explored the impact of an antibody monolayer on the serum matrix effect, since it would favor the binding of high-affinity components—antigens. As matrix interference is usually dependent on diagnostic antibodies,^8^ we have tested three different immunoassays: a direct mouse IgG/anti-mouse IgG, a sandwich human PSA, and a sandwich human IL-1β, covering a range of high-performance immunoassays. We manipulated the antibody surface coverage by varying the concentration of the capture antibody loaded into the microcapillaries, with absorbance responses tested in both buffer and undiluted serum. Surprisingly, we noticed full agreement of optical signals between buffer and undiluted human serum for a narrow range of concentrations of capture antibody (Figure 2), with the window of agreement being very immunoassay-specific.

For the mIgG/anti-mIgG immunoassay (Figure 2A), where both antibodies are polyclonal and do not present site-specific binding, larger antibody surface coverages obtained from 50 to 200 μg/mL mIgG fully eliminated the matrix interference in undiluted serum. Similar results were observed for the IL-1β sandwich assay, where matrix interference was fully eliminated with antibody surface coverages in the range of 40–200 μg/mL (Figure 2B). Based on a previous FEP adsorption study in the same MCF material,^31^ it is known that these CapAb concentrations promote the formation of half of the antibody monolayer with antibodies oriented “end-on” with Fab regions in line, enhancing antigen capture in microcapillary bioassays. This agrees with findings in the literature for a thyroxin assay, where the replacement of an antibody coverage with low affinity by high affinity eliminated the matrix effect of serum samples,^36^ explained by the low-affinity binding of the interfering factor(s) to the immobilized antibody. Consequently, higher antibody coverages with properly oriented antibodies present higher antigen-binding capacity, minimizing sample matrix interference. This is in line with conclusions in another study that reported that matrix proteins bind nonspecifically to the immobilized receptors in IL-6 and acute phase protein (PCT) immunoassays, however not preventing the analyte binding.^37^

The sandwich PSA (Figure 2C), where the immobilized antibody is monoclonal and the detection antibody polyclonal, showed a contrasting behavior, with matrix interference minimized for a narrow window of concentrations (10–20 μg/mL) of the capture antibody, which is significantly lower than for the other antibody systems shown in Figure 2A,B. The polyclonal anti-PSA detection antibody binds directly to the monoclonal CapAb in the absence of the antigen; therefore, an increment in CapAb promotes a higher increment of the signal in buffer than in serum, suggesting a competition of the DetAb with the interfering components from the serum.

These results mean that the narrow window for anti-PSA loading will reduce the assay limit of detection, as sensitivity is linked to the degree of antigen capture, which relates to a higher amount of functionalized antibodies on the surface. Nevertheless, this assay presented the necessary sensitivity for its application, since the PSA clinical threshold is 4 ng/mL.^3^ It is also important to note that optimizing the capture antibody loading in buffer could lead to significant errors in terms of assay performance. In comparison, the IL-1β assay composed of two monoclonal antibodies, showed improved limit of detection since the antibodies are less prone to interference, which is coherent with the general knowledge that assay performance is dependent on antibody nature.^32^

### Matrix Interference is Time-Dependent

**Figure 3:**
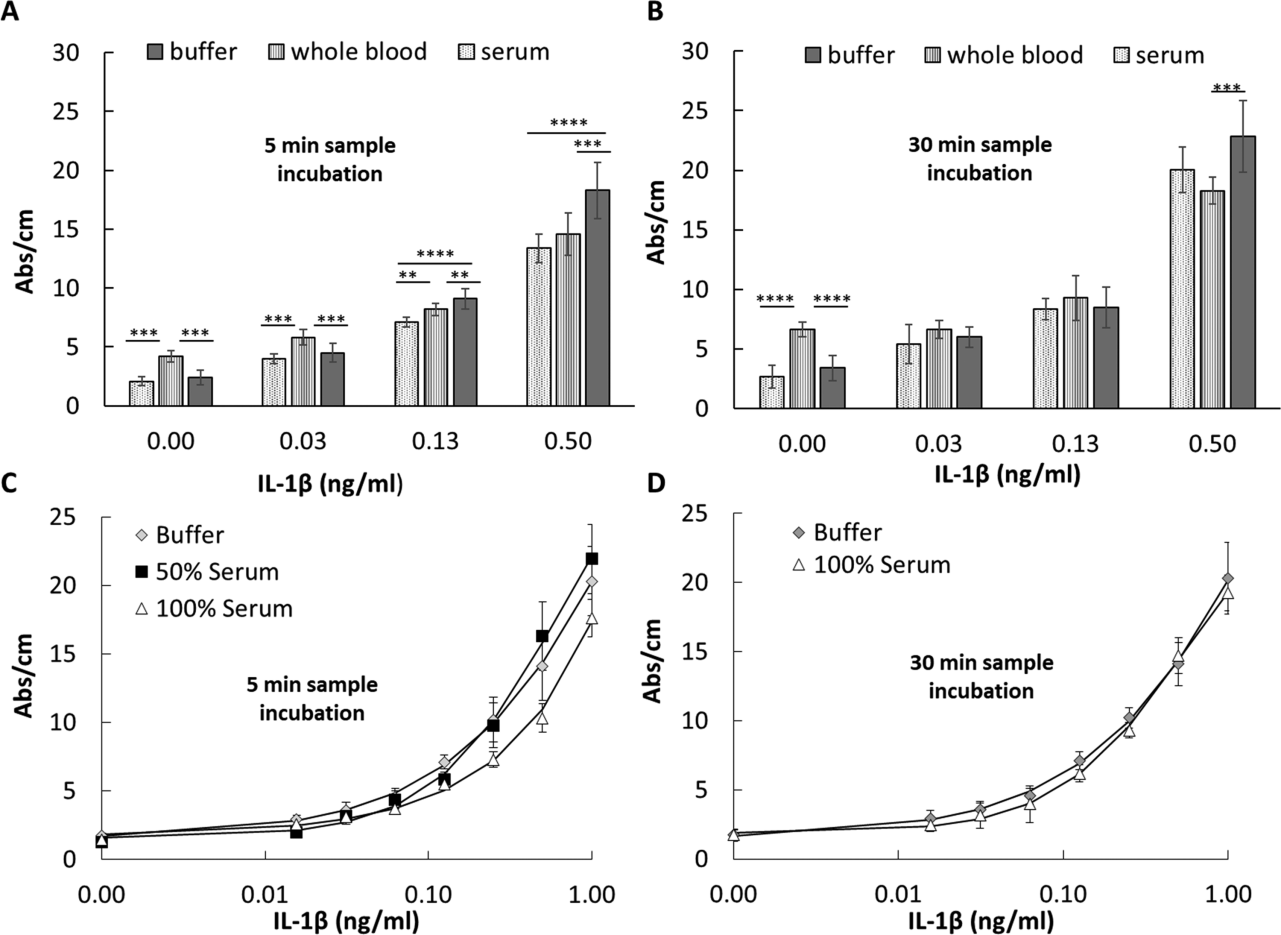
Effect of sample incubation time on IL-1β sandwich MCF immunoassays. (A) IL-1β sandwich assay in buffer, whole blood, and serum, considering 5 min sample incubation time and 0.125 ng/mL IL-1β. (B) IL-1β sandwich assay in buffer, whole blood, and serum, considering 30 min sample incubation time and 0.125 ng/mL IL-1β. (C) MCF IL-1β full response curve using buffer to 50 and 100% human serum as sample diluents. The sample was incubated for 5 min. (D) MCF IL-1β full response curve using buffer and 100% of human serum as sample diluents. The sample was incubated for 30 min. All MCF assays were performed using 40 μg/mL CapAb, which promotes approximately half of the immobilized antibody monolayer with antibodies oriented “end-on” with F(ab) in line.^31^ Note that *P ≤ 0.05; **P ≤ 0.01; ***P ≤ 0.001 in Tukey’s multiple comparisons test.

Longer sample incubation times increase the probability of lower-affinity components to be desorbed and higher-affinity compounds to be bound. In line with our previous experience with the PSA sandwich immunoassay,^3^ where we found a significant impact of sample incubation time on the matrix interference using both whole blood and serum, we have further studied the effect of sample incubation time using a monoclonal pair sandwich assay system. Therefore, we have separately fully tested the effect of sample incubation time and different sample diluents for monoclonal quantitation of IL-1β (Figure 3). Human serum matrix interference was fully eliminated by extending the sample incubation from 5 to 30 min. For whole blood, matrix interference was mostly eliminated for the range of antigen concentrations tested, only with the negative control showing an increase in the background signal (Figure 3A,B). This is undesirable as it impacts the overall limit of detection, yet it can very possibly be addressed through straightforward assay development, such as optimization of the buffer and blocking solutions. Overall, the response curves shown in Figure 3C,D agreed with previous studies with the same PSA sandwich immunoassay^3^ and demonstrated that adequate sample incubation time needs to be combined with suitable antibody surface coverage for minimizing the matrix effect in microcapillary assays. These findings suggest that the matrix interference is time-dependent and very probably linked to a competition for binding sites between low-affinity interfering factor(s) with high-affinity antigens and/or the detection antibody/complex.

A conventional strategy for reducing sample matrix interference in high-sensitivity immunoassays involves diluting the sample, which can be effective depending on the sample dilution factor.^37,38^Figure 3C shows for short incubation time a good overlap of full IL-1β response curves in buffer and 50% human serum, confirming that sample dilution is also effective in capillary immunoassays, agreeing again with our previous results for PSA sandwich immunoassays with human serum and whole blood samples for both colorimetric and fluorescent detection.^3,26^ Yet, with respect to POC applications, sample dilution adds another complex step, which requires automation or precise pipetting and can compromise the clinical value of the test by reducing the limit of detection of the immunoassay. In the case of the IL-1β sandwich immunoassay, we noticed that sample dilution resulted in a similar lower limit of detection (LLoD) in the 50% human serum matrix compared to 100% human serum (Table 1). This supports rapid, high-performance quantitation is also possible in capillary immunoassays with sample dilution. Table 1 also shows that longer incubation times increase LLoDs. LLoDs are determined by the blank value and its standard deviation; therefore, their variation is related to the development of background noise, which can be caused by nonspecific binding of serum/blood components. Although longer periods increase the probability of desorption of low-affinity components in the presence of the analyte eliminating the matrix effect, they increase the probability of nonspecific binding in the absence of the analyte, negatively impacting the LLoD of assays. Consequently, it is important to find a suitable sample incubation time that can simultaneously enable the management of the matrix effect and maintain the desired LLoD performance.

**Table 1:** IL-1β Sandwich Assay Sensitivity Considerations in Buffer and in Human Serum after 4PL Model Fitting and Analyses (Figure 3C,D)

sample incubation time (min)	sample matrix	lower limit of detection (LLoD) (ng/mL)	precision (with 0.125 ng/mL IL-1β) (%)	*R*^2^ (with 4PL model)
5	buffer	0.021	9	0.9992
5	100% serum	0.014	6	0.9989
5	50% serum	0.006	19	0.9991
30	buffer	0.084	20	0.9929
30	100% serum	0.051	10	0.9965

### Diameter Dependence of the Matrix Effect

**Figure 4:**
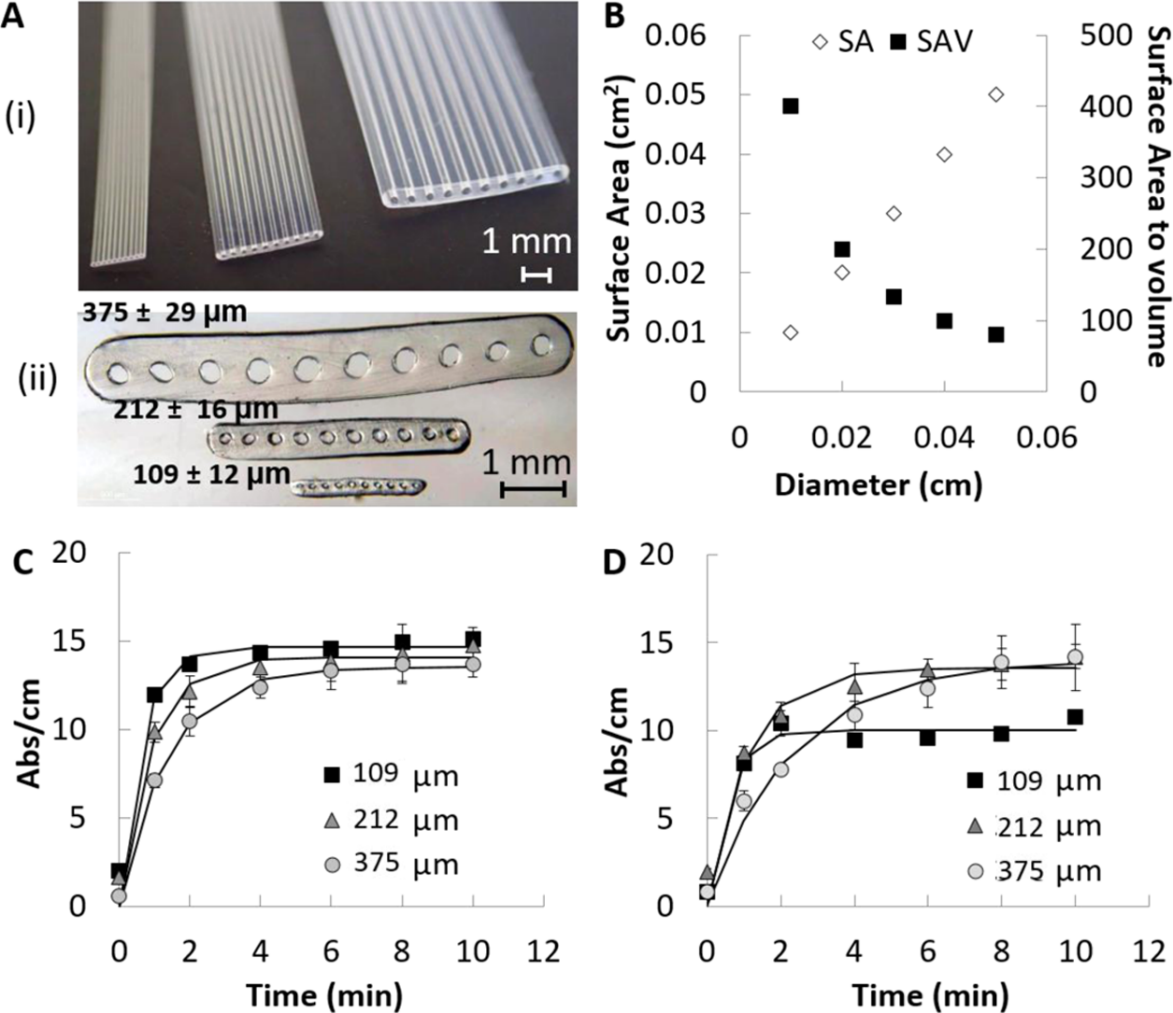
Relationship between the human serum matrix effect and capillary geometry in MCF assays. (A) (i) Photograph of three different MCFs with 109 ± 12, 212 ± 16, and 375 ± 29 μm mean diameter bore. (ii) Microscopy photograph of a cross section from the MCFs with 109, 212, and 375 μm diameter bore. (B) Effect of diameter size on the total surface area (SA) and on the surface-area-to-volume ratio (SAV). (C) mIgG–anti-mIgG binding kinetics on different diameter MCFs in the buffer matrix. (D) mIgG–anti-mIgG binding kinetics in different diameter MCFs in the undiluted human serum matrix.

We have recently reported that surface coverage of an antibody by passive adsorption in Teflon FEP microfluidic strips is dependent on the capillary diameter^31^ (Figure 4A). From a theoretical perspective, the capillary diameter is known to affect the total surface area (SA) available for antibody immobilization, as well as the sample/reagent volume (*V*), which in turn affects the mass and density of the immobilized capture antibody. On the other hand, the surface-area-to-volume ratio (SAV) becomes an important parameter as it can govern the antigen–antibody equilibrium and the rate of the reaction rate; overall the choice about the capillary diameter can be seen as a balance between the total SA and the SAV (Figure 4B). Although surface density (ng/cm^2^) is independent of the diameter of the capillary, due to the smaller sample volume loaded, small diameter capillaries yield a much lower surface density of the immobilized antibody compared to larger diameter capillaries. In such a case, the number of adsorbed molecules is limited by the number of molecules in solution. Barbosa et al.^31^ reported half of the amount of immobilized antibodies on the 109 μm diameter MCF compared to the amount immobilized on MCF strips with mean internal diameter 212 μm. On the other hand, the 375 μm diameter MCF also presented a significantly higher maximum surface density compared to the 212 μm MCF (867.8 and 609.5 ng/cm^2^, respectively). Capillary diameter also affects the maximum diffusion distance that molecules have to travel in a heterogeneous immunoassay (with the capture antibody or first member of the binding pair immobilized on the inner wall of the capillaries) with the time of diffusion increasing to the square of the distance according to Einstein’s law of diffusion.^39^ Diffusion can be affected by the viscosity of the sample; however, immunoassay signals with serum samples were not significantly different than signals with the buffer and we have not detected any significant variations in both assay kinetics and equilibrium, as shown and discussed in the Supporting Information (Figure S1 and Table S1). Consequently, we have studied the effect of capillary diameter in MCF assays with the mIgG/anti-mIgG assay. We noticed a decrease in capillary diameter from 212 to 109 μm in buffer resulted in 2 min faster antibody–antigen equilibrium, while an increase in capillary diameter from 212 to 375 μm delayed the equilibrium by 2 min, confirming that capillary immunoassays are also diffusion-limited (Figure 4C and Table 2). Note that kinetic constants obtained reflect the overall strength and stability of the antibody monolayer, which depends on structural rearrangements of mIgG antibodies and the fact that anti-mIgG can bind in a bivalent way to the immobilized antibodies if properly oriented. Also, the low-affinity antigen, like anti-mIgG, is strongly affected by the mIgG density and therefore structural orientation.^40^ Further experiments with undiluted human serum showed that the equilibrium is surprisingly changed for small capillaries (Figure 4D and Table 2) in the presence of the biologic human serum matrix, revealing a level of interference of the matrix that could not be detected by comparing other microcapillary diameters tested. This can be explained by the different antibody surface coverages in the different capillary geometries due to the adsorption equilibrium and on/off rates of the immobilized antibody onto Teflon FEP surfaces being dependent on capillary geometry, as explained previously. Overall, this confirmed a correlation between antibody surface coverage, microcapillary diameter, and matrix interference, with reduced antibody coverages being more prone to matrix interference.

**Table 2:** Kinetic Constants of Anti-mIgG Binding in Buffer and Human Serum in Different Capillary Diameter MCFs (Figure 4C,D)

	buffer			human serum		
	109 μm MCF	212 μm MCF	375 μm MCF	109 μm MCF	212 μm MCF	375 μm MCF
*K*_on_ (M s^–1^)	6.39 × 10^6^	4.24 × 10^6^	2.65 × 10^6^	5.38 × 10^6^	3.35 × 10^6^	9.62 × 10^7^
*K*_off_ (s^–1^)	1.42 × 10^–3^	1.71 × 10^–3^	1.55 × 10^–3^	8.52 × 10^–3^	1.91 × 10^–3^	4.13 × 10^–2^
*K*_d_ (M^–1^)	2.23 × 10^–10^	4.04 × 10^–10^	5.87 × 10^–10^	1.58 × 10^–9^	5.70 × 10^–10^	4.29 × 10^–10^

### Novel Analytical Approach for Managing Matrix Interference in Miocrocapillary Protein Immunoassays

**Figure 5:**
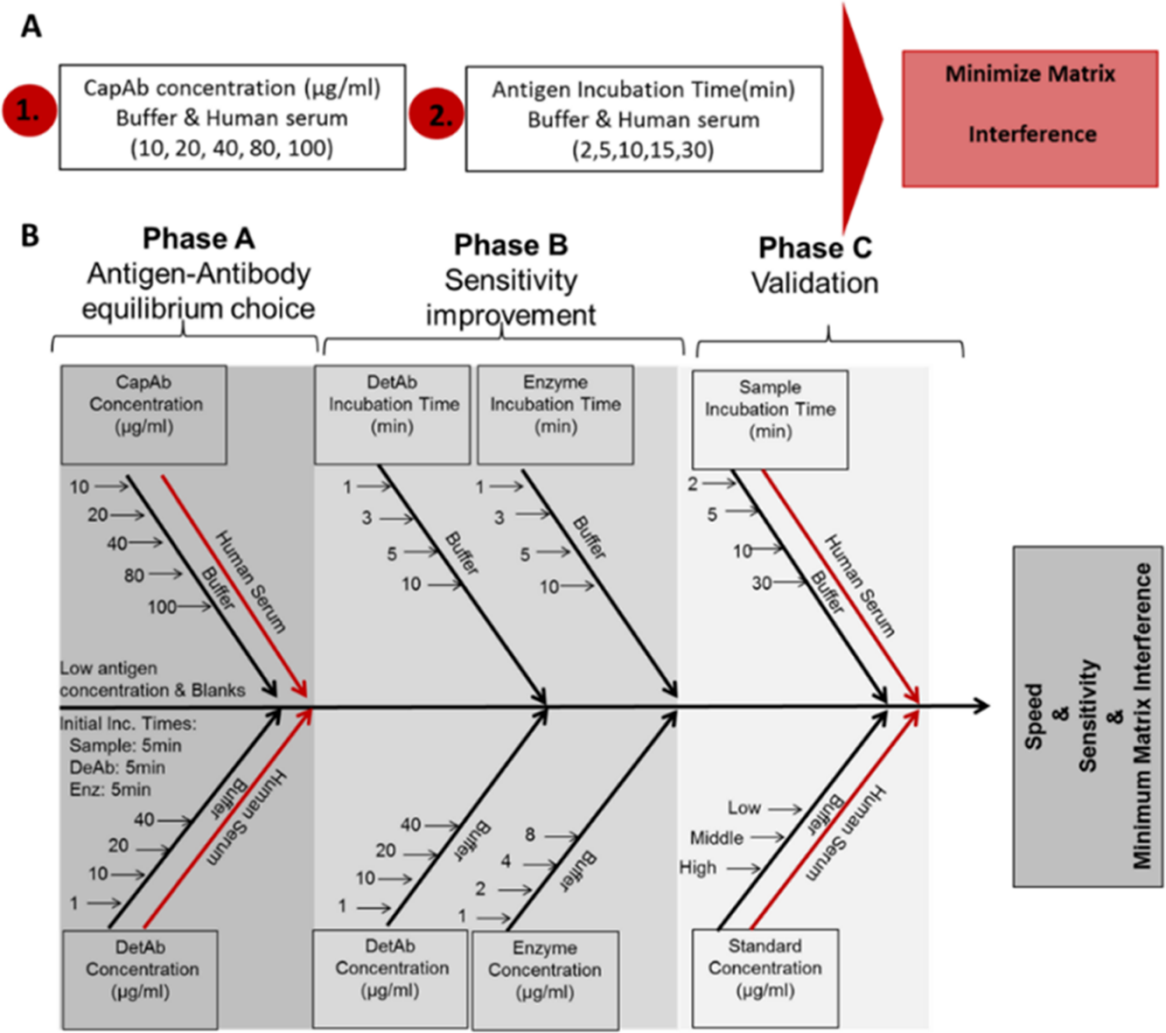
Analytical approach for minimizing biological matrix interference in MCF sandwich assays. (A) Diagram with CapAb concentration and sample incubation time for minimizing matrix interference in MCF assays. (B) Diagram showing MCF assay development and optimization for rapid, sensitive, and accurate quantitative assays.

We have combined the new finding in sample matrix interference with our several years’ experience in high-performance microcapillary immunoassays to propose a new analytical approach (Figure 5A), which we believe will help the effective management of the sample matrix effect in miniaturized immunoassays. This approach can easily be integrated into routine assay development helping to deliver more robust microfluidic immunoassays, especially in the MCF platform, enabling rapid, sensitive, accurate, and decentralized quantitative protein immunoassay testing (Figure 5B). The first stage in the development of, e.g., a protein sandwich immunoassay should be the choice of optimal antibody surface coverage for minimal matrix interference. This can be done by changing CapAb concentration in buffer, yet it is essential that this is also carried out in human serum. Low antigen concentrations will favor the antibody surface coverage yielding an enhanced limit of detection for the test (Phase A, Figure 5). For the best signal-to-noise ratio (yielding the lowest limit of detection), concentration and incubation times should be optimized for both the detection antibody and enzyme. This assay development stage can be performed in buffer (Phase B, Figure 5) and easily translated into whole serum. Finally, the protein immunoassay should be performed in buffer and serum samples, with sample incubation times varied to obtain the effective working window offering negligible or minimum matrix interference (Phase C, Figure 5). This integrated analytical approach will enable the accurate quantitation of different proteins in the microfluidic platforms from nondiluted serum samples, as shown in this work.

## Conclusions

Human serum sample matrix interference was fully eliminated in three different miniaturized enzymatic immunoassays (a direct mIgG/anti-mIgG, a sandwich human PSA, and a sandwich human IL-1β) by manipulating antibody surface coverage and sample incubation time. An optimal antibody density, with antibodies presenting optimal binding capacity, is ideal for overcoming the matrix effect. Longer sample incubation can be effective in minimizing sample interference for certain capillary immunoassays, with equilibrium clearly not affected by the matrix effect, only the kinetics of binding being slowed down, yet the strategy revealed diameter-dependent. Our results pointed to matrix interference being linked to a competition between low-affinity interference factor(s) and high-affinity antigens and reagents; therefore, both sample dilution and incubation time are effective in minimizing matrix interference in microcapillary immunoassays. The novel simple, analytical immunoassay development approach proposed is expected to help in speeding up the development of robust, accurate, high-performance, and decentralized miniaturised protein immunoassays. The results shown are perhaps specific to fluoropolymer microcapillaries (which allow production of hundreds or thousands of disposable test strips at a minimum cost without any complex automation), yet the surface properties of Teflon FEP are not so distinct from other polymers such as PDMS (both hydrophobic); therefore, we believe that these new bioanalytical approaches can benefit both the research and innovation communities working on immunoassay miniaturization including conventional and modern microfluidic technologies for POC testing.

## Supporting Information Available

Sourcing of the MCF material; effect of sample viscosity; kinetics of antibody binding; effect of antibody surface coverage; statistical analysis; and image analysis of MCF strips (PDF)
