# Supplementary material for: Antibody Surface Coverage Drives Matrix Interference in Microfluidic Capillary Immunoassays
Source: ACS Sens. 2021 Jun 17;6(7):2682–90. doi: 10.1021/acssensors.1c00704 (PMC8741144; doi:10.1021/acssensors.1c00704)
Supplement: Supplementary file 1 — se1c00704_si_001.pdf [file se1c00704_si_001.pdf]

Supporting Information for:

Antibody surface coverage drives matrix interference in microfluidic capillary immunoassays

Ana I. Barbosa[†,#], Alexander D. Edwards[$,#], and Nuno M. Reis[¥,#,*]

[†]Department of Chemical Engineering, Loughborough University, Loughborough, LE11 3TU, United Kingdom, [#]Capillary Film Technology Ltd, Daux Road, Billingshurst, RH14 9SJ West Sussex, United Kingdom, [$]Reading School of Pharmacy, University of Reading, Whiteknights, Reading RG6 6AD, United Kingdom, [¥]Department of Chemical Engineering and Centre for Biosensors, Bioelectronics and Biodevices (C3Bio), University of Bath, Claverton Down, Bath BA2 7AY, United Kingdom

Table of Contents:

Supplementary methods	pp S2
Supplementary results and discussion	pp S4
Table S1	pp S4
Figure S1	pp S5
Supplementary references	pp S6

[*] **Corresponding author:** N. M. Reis. E-mail: n.m.reis@bath.ac.uk; Tel.: +44(0)1225 383 369.

SUPPLEMENTARY METHODS

Microfluidic fluoropolymer MCF strips. The MFC materials used in this study were fabricated from fluorinated ethylene propylene co-polymer (Teflon® FEP) by melt-extrusion by Lamina Dielectrics Ltd. (Billinghurst, West Sussex, UK), presenting 10 parallel microcapillaries with mean hydraulic diameters of 109±12 µm, 212±16 µm, and 375± 29 µm. We have recently published isotherms and kinetics of antibody adsorption onto these same MCF materials.[1]

Effect of sample viscosity. This was done using IL-1β sandwich immunoassay which followed the protocol described previously,[29] however in the present work the effect of matrix viscosity, surface coverage and sample incubation time, either in buffer, human serum, and whole blood are showed for the first time. Briefly, the MCF strips used were 212±16 µm bore diameter, and 40 µg/ml of IL-1β capture antibody concentration was used to promote half of an antibody monolayer coverage.[27] Three different solutions of BSA with concentrations of 5, 10 and 25 w/v % were used as sample diluents for 1 ng/ml and 0.1 ng/ml of IL-1β, providing the IL-1β antigen with different viscosities medium. For more details see SI.

For viscosity measurement of sample, a U-tube viscometer, size B (nominal constant = 0.01cSt/s), supplied from Technico, was used to measure kinematic viscosity (cSt or 1 mm^2/s) of PBS, BSA (5, 10 and 25%) and human serum dilution solutions. The solutions were filled into a tube in a water bath with temperature control set at 20ºC. The time (seconds) for the liquid to move from distance X to Y in the U-tube was measured 3 times for each sample. The specific mass for each sample was determined by weighing the sample liquid into a vial and determining the volume of the vial by weighing distilled water, assuming a specific mass for water of $1g/cm^3$. Each measurement was performed 3 times in a temperature controlled room. The dynamic viscosity (μ) in mPa.s was obtained by multiplying the kinematic viscosity of the sample in cSt ($=1E^{-6}$ m^2/s) by the sample density in kg/dm^3.

Eight MCF strips with 212±16 µm bore diameter were loaded with 40 µg/ml of IL-1β CapAb and incubated for 2h at room temperature. This solution was replaced by the super blocking solution, incubated for an additional 2h at room temperature. The MCF strips were washed with 1 ml of PBS-Tween, and trimmed into 30 mm length test strips. The test strips were introduced into the Multiple Syringe Aspirator (MSA), a fluid handling device that allows simultaneous aspiration of solutions into eight different MCF strips, presented elsewhere.[2] Three different solutions of BSA with concentrations of 5, 10 and 25 w/v % were used as sample diluents for 1 ng/ml and 0.1 ng/ml of IL-1β. These solutions were aspirated into the coated and blocked MCF strips connected to the MSA and left to incubate for variable incubation times. After a washing step, a monoclonal anti-IL-1β biotinylated DetAb (10 µg/ml) incubated for 10 min followed by another washing step. A solution of 4 µg/ml of high sensitivity streptavidin horseradish peroxidase (HRP) was aspirated into the capillaries and incubated for 10 min followed by an intensive washing step. Finally, a 4 mg/ml solution of OPD enzymatic substrate replaced the washing buffer and was incubated for 5 min.

Effect of antibody surface coverage. In the mIgG/anti-mIgG system eleven MCF strips, with 212 µm diameter and 8 cm length, were filled with different solutions of mouse IgG in the range of 0 -200 µg/ml. These solutions were incubated for 30 minutes at room temperature, followed by a wash step. The MCF strips were trimmed into 3 cm strips and were placed in the Multiple Syringe Aspirator (MSA). A solution of 0.6 µg/ml of anti-mIgG peroxidase conjugated, prepared in PBS buffer, replaced the wash buffer and was incubated for 10 minutes, followed by another washing step. The OPD enzymatic substrate (1 mg/ml) was aspirated into the MCF strips and the MSA was placed on the flatbed scanner. Digital images were taken after 5 minutes of enzymatic substrate incubation time. The same procedure was repeated for anti-IgG solutions prepared in 100% human serum. For IL-1β sandwich immunoassay, eight MCF strips with 212 µm diameter and 16 cm length, were filled with different solutions of monoclonal anti-IL-1β (0-200 µg/ml range). A solution of 0.125 ng/ml of IL-1β was prepared in PBS buffer and human serum and incubated for 30 minutes. The following steps are described above. In the PSA sandwich immunoassay eight MCF strips, with 212 µm diameter and 8 cm length, were filled with eight different solutions of monoclonal anti-PSA and incubated for 2 hours at room temperature. The strips were then filled with 3% BSA solution which was also incubated inside the capillaries for 2 hours at room temperature, followed by a washing step. The MCF strips were trimmed into 30 mm long test strips and placed in the MSA. A solution of 3.75 ng/ml of PSA standard was prepared in PBS buffer, aspirated into the MCF strips and incubated for 30 minutes, followed by a wash step. A polyclonal biotinylated detection antibody (DetAb) (1 µg/ml) was incubated for 5 minutes, followed by 5 min incubation of high sensitivity streptaving peroxidase (1 µg/ml). An intensive washing step was performed, and OPD enzymatic substrate (4 mg/ml) filled the MCF test strips. The MSA was placed on the flatbed scanner and digital images were taken in 5 minutes of enzymatic substrate incubation. The procedure was repeated for PSA standards prepared in 100% human serum.

Statistical analysis. To determine statistical significance, data were analyzed using GraphPad Prism v5.01 software (GraphPad Software, CA). All statistics were reported as mean ± standard deviation. One-way ANOVA analysis was used, by applying Tukey multiple comparisons test. The immunoassays were performed with n=10, and statistical significance was considered at $p < 0.05$.

Image analysis of the microfluidic MCF strips. RGB digital images of the immunoassay strips were split into 3 separated channels images by *Image J* software (NIH, Maryland, USA). The blue channel images were used to calculate absorbance values, based on the grey scale peak height of each individual capillary of TEFLON® FEP-Teflon MCF as described previously.[2,4] Therefore, absorbance signal is calculated for each capillary, according Beer-Lambert equation. The absorbance values presented averages of absorbance from 10 capillaries in a given MCF stip.

SUPPLEMENTARY RESULTS AND DISCUSSION

Effect of sample viscosity. Biological samples present different viscosity to an immunoassay buffer, which can affect the time for antigen diffusion, interfering with the kinetics of antibody-antigen, and introducing variability in the immunoassay. Sample dilution is a very standard strategy for minimising matrix interference, with some studies showing viscosity in the biological sample as the major interfering factor in immunoassays.[31] In order to clarify the role of sample viscosity in capillary immunoassays, we carried out a full sandwich IL-1β sandwich protein assay with IL-1β protein spiked in buffer with different concentrations of BSA, yielding a range of dynamic viscosities (Figure S1). Though we noticed a delay on the binding of IL-1β, that effect was only detectable for 25% w/w of BSA (Figure S1A), corresponding to 250 mg/ml BSA with a dynamic viscosity of 3.51 mPa.s, which is about double of viscosity of undiluted human serum (Figure S1B). Interestingly, the equilibrium of the reaction (Figure S1A and Table S1) was not dependent on the BSA concentration nor viscosity. The delay in equilibrium was not observed for lower antigen concentrations (Table S1, in this case 0.1 ng/ml of IL-1β). This means sample dilution or extension of sample incubation times (as reported in previous studies[11,24]) help dealing with diffusion limitations due to viscosity of the matrix yet these do not directly address the root cause of sample interference.

Table S1 - Kinetic constants of IL-1β binding for 1 and 0.1 ng/ml of IL-1β shown in Figure S1A. Kinetic constants obtained from fitting experiomental data to equation (1) shown in main document

	**1 ng/ml IL-1β**				**0.1 ng/ml IL-1β**			
	**PBS**	**50 mg/ml BSA**	**100 mg/ml BSA**	**250 mg/ml BSA**	**PBS**	**50 mg/ml BSA**	**100 mg/ml BSA**	**250 mg/ml BSA**
K_{on} (M.s^{-1})	2.36×10^{15}	3.07×10^{11}	2.70×10^{11}	9.56×10^{10}	1.79×10^{11}	2.86×10^{11}	2.30×10^{11}	1.08×10^{11}
K_{off} (s^{-1})	1.11×10^{-1}	1.75×10^{-3}	1.09×10^{-3}	8.88×10^{-4}	3.81×10^{-4}	1.91×10^{-3}	3.37×10^{-3}	5.57×10^{-4}
K_A (M)	2.13×10^{14}	1.76×10^{14}	2.48×10^{14}	1.08×10^{14}	4.70×10^{14}	1.49×10^{14}	6.83×10^{13}	1.93×10^{14}

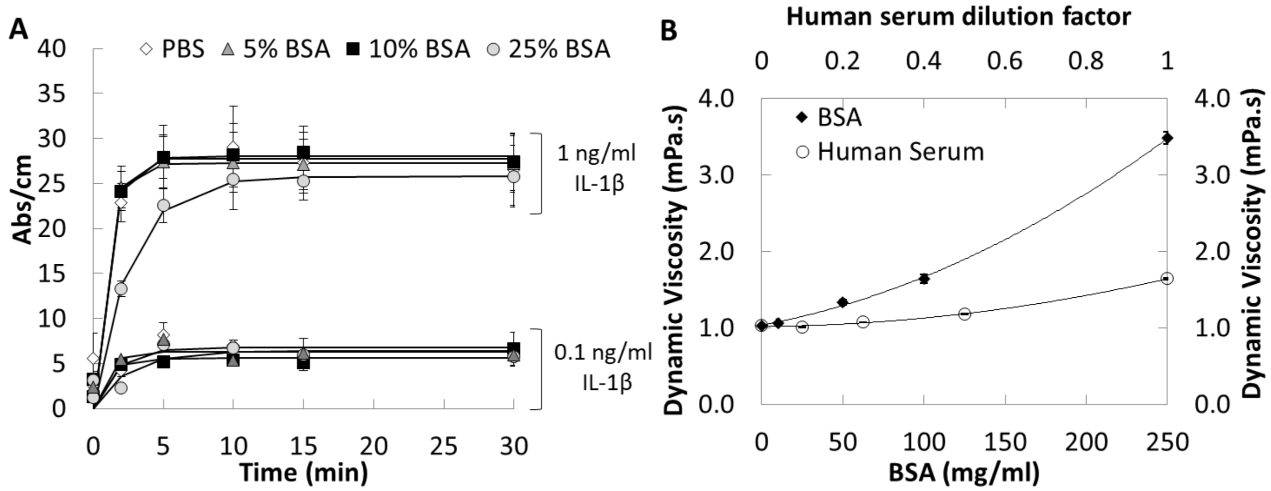

Figure S1. Effect of sample matrix viscosity on MCF IL-1β sandwich assay, using 212 µm diameter bore MCF. A Antibody-antigen (IL-1β 0.1 and 1 ng/ml) kinetics with different sample diluents: PBS, 50, 100 and 250 mg/ml BSA. B Viscosity of the BSA solutions used and viscosity of human serum different dilutions.

Supplementary References

(1) Barbosa, A. I.; Barreto, A. S.; Reis, N. M. Transparent, Hydrophobic Fluorinated Ethylene Propylene Offers Rapid, Robust, and Irreversible Passive Adsorption of Diagnostic Antibodies for Sensitive Optical Biosensing. *ACS Appl. Bio Mater.* **2019**, *2* (7), 2780–2790. https://doi.org/10.1021/acsabm.9b00214.

(2) Barbosa, A. I.; Castanheira, A. P.; Edwards, A. D.; Reis, N. M. A Lab-in-a-Briefcase for Rapid Prostate Specific Antigen (PSA) Screening from Whole Blood. *Lab Chip* **2014**, No. 14, 2918–2928. https://doi.org/10.1039/c4lc00464g.

(3) Zimmermann, M.; Delamarche, E.; Wolf, M.; Hunziker, P. Modeling and Optimization of High-Sensitivity, Low-Volume Microfluidic-Based Surface Immunoassays. *Biomed. Microdevices* **2005**, *7* (2), 99–110. https://doi.org/10.1007/s10544-005-1587-y.

(4) Edwards, A. D.; Reis, N. M.; Slater, N. K. H.; Mackley, M. R. A Simple Device for Multiplex ELISA Made from Melt-Extruded Plastic Microcapillary Film. *Lab Chip* **2011**, *11* (24), 4267–4273. https://doi.org/10.1039/C0LC00357C.
